# Hybrid Organic-Inorganic Perovskite Memory with Long-Term Stability in Air

**DOI:** 10.1038/s41598-017-00778-5

**Published:** 2017-04-06

**Authors:** Bohee Hwang, Jang-Sik Lee

**Affiliations:** Department of Materials Science and Engineering, Pohang University of Science and Technology (POSTECH), Pohang, 790-784 Korea

## Abstract

Organic-inorganic perovskite materials have attracted extensive attention for wide range of applications such as solar cells, photo detectors, and memory devices. However, the lack of stability in ambient condition prevented the perovskite materials from applying to practical applications. Here, we demonstrate resistive switching memory devices based on organic-inorganic perovskite (CH_3_NH_3_PbI_3_) that have been passivated using thin metal-oxide-layers. CH_3_NH_3_PbI_3_-based memory devices with a solution-processed ZnO passivation layer retain low-voltage operation and, on/off current ratio for more than 30 days in air. Passivation with atomic-layer-deposited (ALD) AlO_x_ is also demonstrated. The resistive switching memory devices with an ALD AlO_x_ passivation layer maintained reliable resistive switching for 30 d in ambient condition, but devices without the passivation layer degraded rapidly and did not show memory properties after 3 d. These results suggest that encapsulation with thin metal-oxide layers is easy and commercially-viable methods to fabricate practical memory devices, and has potential to realize memory devices with long-term stability and reliable, reproducible programmable memory characteristics.

## Introduction

Organic-inorganic perovskite (OIP) materials are widely used in electronic and optoelectronic devices including light-emitting diodes^1, 2^, photo detectors^3, 4^, and lasers^5, 6^ and in solar cells^7, 8^. OIPs contain defects, which migrate when subjected to an electrical field; as a result this material exhibits sweep-dependent hysteresis in current-voltage (*I* − *V*) responses^9^. This ion migration in a perovskite layer can form a reversible p-i-n structure, in which photocurrent direction can be switched by applying a small electric field^10^. Moreover, organic cations that can rotate under an applied electrical field show ferroelectric behavior by positive and negative poling, and have structural flexibility^11^. These properties of OIP materials suggest applications as computer memory application^12–16^.

However, OIPs are not stable in humidity and ambient atmosphere, so devices break down quickly^17^. For this reason, OIP films should be fabricated in N_2_ atmosphere, and devices that are not encapsulated cannot last long in the ambient atmosphere^18, 19^; this characteristic impedes commercialization and application of OIP electronic devices. To improve the long-term stability of OIP solar cells, various approaches have been tested. For example, ultrathin Al_2_O_3_ layers on the OIP layer isolate the perovskite layer from moisture, and thereby increase device stability^20^. Hydrophobic oligothiophene hole transport layers (HTLs) have been used as a protective layer for OIP film^21^. Solution-processed ZnO nanoparticle (NP) film that functions as an electron transport layer improved the efficiency and the stability of the cell^22^. However, the effect of passivating OIP films for memory applications has not been studied.

In this study, we selected ZnO and AlO_x_ as the protecting layer to protect OIPs from degradation by moisture and air. ZnO is already used as an air-stable cathode in polymer light emitting diodes^23^, and has been applied in solar cells as charge transport layer that also shields the photoactive layer from the ambient air^24^. Chemically-modified ZnO nanorods are water- resistant due to nanostructures with low surface energies that yield high contact angles with water droplets^25^. Al_2_O_3_ layers fabricated using atomic layer deposition (ALD) have been used as protective coatings for copper^26^, and as gas-diffusion barriers for polymer substrates^27^. These features may protect the perovskite device from moisture. We fabricated air-stable OIP (CH_3_NH_3_PbI_3_)-based ReRAM devices passivated by metal-oxide layers that are deposited by different methods such as solution process and ALD process. Devices without the passivation layer degraded after exposure to ambient air for less than 3 d. In contrast, all-solution-processed Au/ZnO/CH_3_NH_3_PbI_3_/ITO memory devices showed reliable operation for 30 d in ambient air, and Al/ALD_AlO_x_/CH_3_NH_3_PbI_3_/ITO memory devices showed bipolar resistive switching property for 30 d in ambient air. This concept of with metal-oxide layer passivation could realize perovskite memory devices that work stably in ambient air.

## Results and Discussion

**Figure 1:**
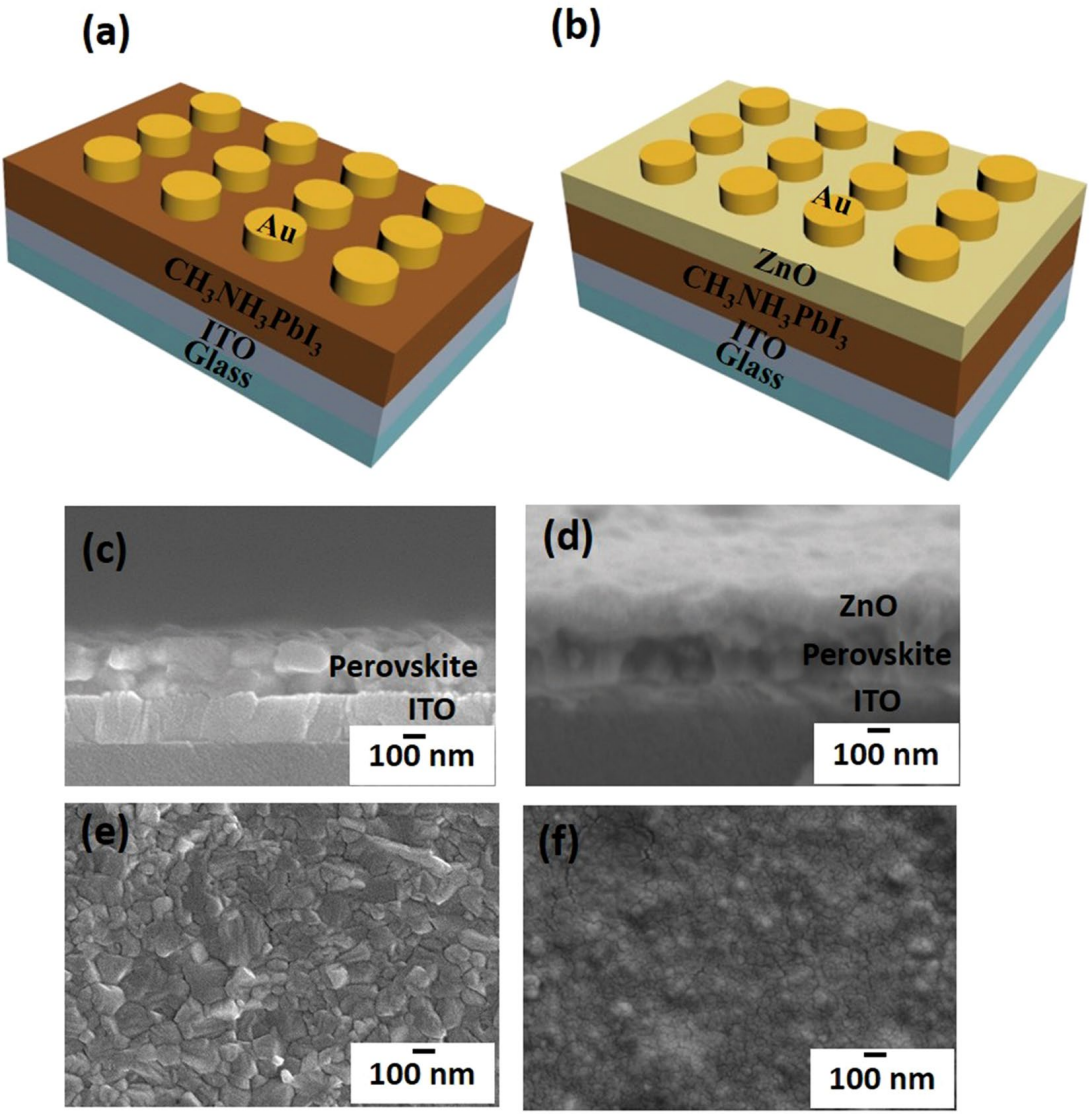
Schematic of (**a**) Au/CH_3_NH_3_PbI_3_/ITO devices and (**b**) Au/ZnO/CH_3_NH_3_PbI_3_/ITO devices. Cross-sectional images of (**c**) Au/CH_3_NH_3_PbI_3_/ITO devices and (**d**) Au/ZnO/CH_3_NH_3_PbI_3_/ITO devices. Plan views of perovskite (**e**) without ZnO film and (**f**) with ZnO film.

Au/CH_3_NH_3_PbI_3_/ITO-coated glass and Au/ZnO/CH_3_NH_3_PbI_3_/ITO-coated glass were used to demonstrate memory devices that have a metal/insulator/metal structure (Fig. 1a,b). A two-step spin coating method^28^ was used to coat CH_3_NH_3_PbI_3_ layer on a PbI_2_ surface. The CH_3_NH_3_PbI_3_ layer synthesized on ITO-coated glass formed a uniform film of thickness ~218 nm (Fig. 1c). The ZnO permeation barrier was then formed by repeating spin-coating of ZnO NPs (dispersed in chlorobenzene) on the CH_3_NH_3_PbI_3_ layer^22, 29^. Photomicrographs of the perovskite film with ZnO NPs (Au electrode not included) (Fig. 1d) show individual layer of ZnO (105 nm thick) and CH_3_NH_3_PbI_3_ (196 nm thick). The perovskite film without ZnO layer consisted of dense and closely-packed grains with the sizes of 100–200 nm. (Fig. 1e), and the perovskite film capped by ZnO layer was homogenously covered with ZnO NPs (Fig. 1f).

**Figure 2:**
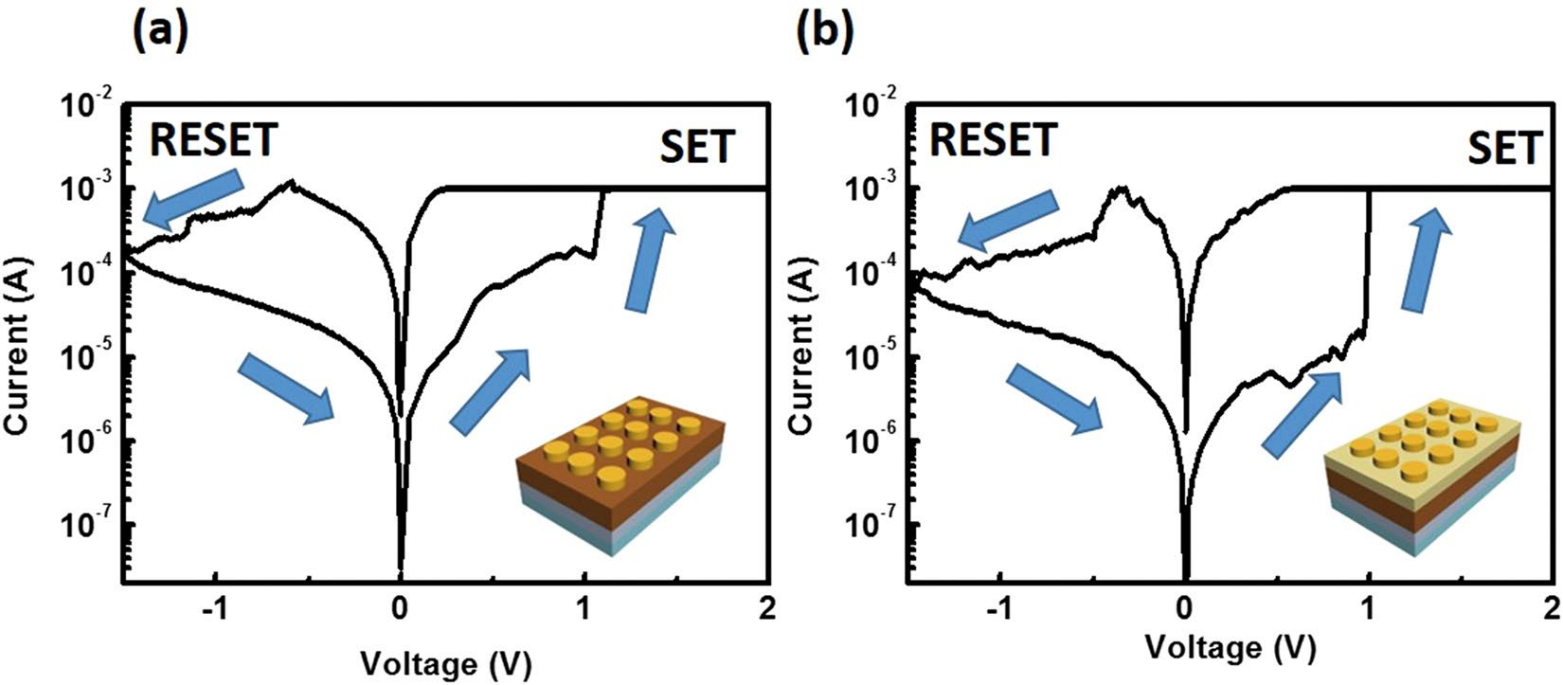
Resistive switching characteristics of hybrid OIP based devices. (**a**) Au/CH_3_NH_3_PbI_3_/ITO/glass devices and (**b**) Au/ZnO/CH_3_NH_3_PbI_3_/ITO/glass devices.

The electrical properties of Au/CH_3_NH_3_PbI_3_/ITO and Au/ZnO/CH_3_NH_3_PbI_3_/ITO devices were characterized under ambient conditions; in both devices, the measured *I* − *V* curves exhibited bipolar resistive switching under compliance current of *CC* = 1 mA (Fig. 2a,b). To measure the *I* − *V* characteristics of the Au/CH_3_NH_3_PbI_3_/ITO device and the Au/ZnO/CH_3_NH_3_PbI_3_/ITO device, the voltage was controlled by one of the Au electrodes under dc sweeping voltage applied as 0 V → 2 V → 0 V → −1.5 V → 0 V; the bottom electrode (ITO) was grounded. In the CH_3_NH_3_PbI_3_-based device without ZnO layer, during the first voltage sweep from 0 V to set voltage *V*_set_~1.1 V, positively-charged iodine vacancies migrate toward the negatively-charged electrode (ITO) to form conductive filaments that transport carriers injected from electrodes^15^. After the conductive filaments formed, the resistance state changed from high-resistance state (HRS) (OFF state) to low-resistance state (LRS) (conductive ON state). When a negative voltage was applied, the current decreased gradually at reset voltage *V*_reset_ < −0.6 V, and the resistance changed from LRS to HRS (Fig. 2a). To quantify the switching speed of CH_3_NH_3_PbI_3_, we measured the pulse width of the set and reset voltages (Figure S1). The CH_3_NH_3_PbI_3_-based memory device switched quickly under a voltage pulse. A dc voltage bias sweep (0 to 0.3 V) was applied to measure the device state before the pulse was applied to measure the device state before the pulse was applied. The voltage bias sweep will not result in resistive switching due to failure of set and reset processes in this voltage range. After using a pulse generator to apply a pulse, dc voltage bias sweep was used to determine the resistance change of the device. A set voltage pulse (+2 V, 1 µs) and a reset voltage pulse (−2 V, 1 µs) were applied to switch the device resistance state. A 1-µs pulse width was sufficient to change the resistance state of devices. The CH_3_NH_3_PbI_3_-based memory devices switched in <10 µs, which is competitive with conventional flash memory specifications.

The electrical characteristics of Au/ZnO/CH_3_NH_3_PbI_3_/ITO device were examined using the same voltage sweep and measurement method. The device with passivated ZnO layer also showed bipolar resistive switching behavior. The device that was passivated with ZnO had *V*_set_~0.9 V, which is similar to *V*_set_ of the device without ZnO.

The on/off current ratio *I*_ON_/*I*_OFF_ of the device with ZnO was slightly higher than that of the device without ZnO, however, the electrical properties of Au/CH_3_NH_3_PbI_3_/ITO and Au/ZnO/CH_3_NH_3_PbI_3_/ITO devices were similar, which indicated that ZnO layer did not influence the resistive switching behavior. The data retention property was evaluated to test the stability of the memory device encapsulated with ZnO layer with a reading voltage of 0.2 V under ambient conditions (Figure S2a). The Au/ZnO/CH_3_NH_3_PbI_3_/ITO device maintained constant *I*_ON_/*I*_OFF_ for 10^4^ s. In a repeated cyclic test, the electrical characteristic of the device showed almost not change over 100 cycles (Figure S2b). The cycling endurances of Au/ZnO/CH_3_NH_3_PbI_3_/ITO devices were measured using consecutive ac voltage pulses to evaluate the electrical stability under *V*_*se*t_ = +2 V and *V*_*reset*_ = −2 V. (Figure S2c) The width of the voltage pulse was 10 ms and the read voltage was 0.2 V. The endurance properties varied over time, but neither LRS nor HRS degraded for up to 50 cycles.

**Figure 3:**
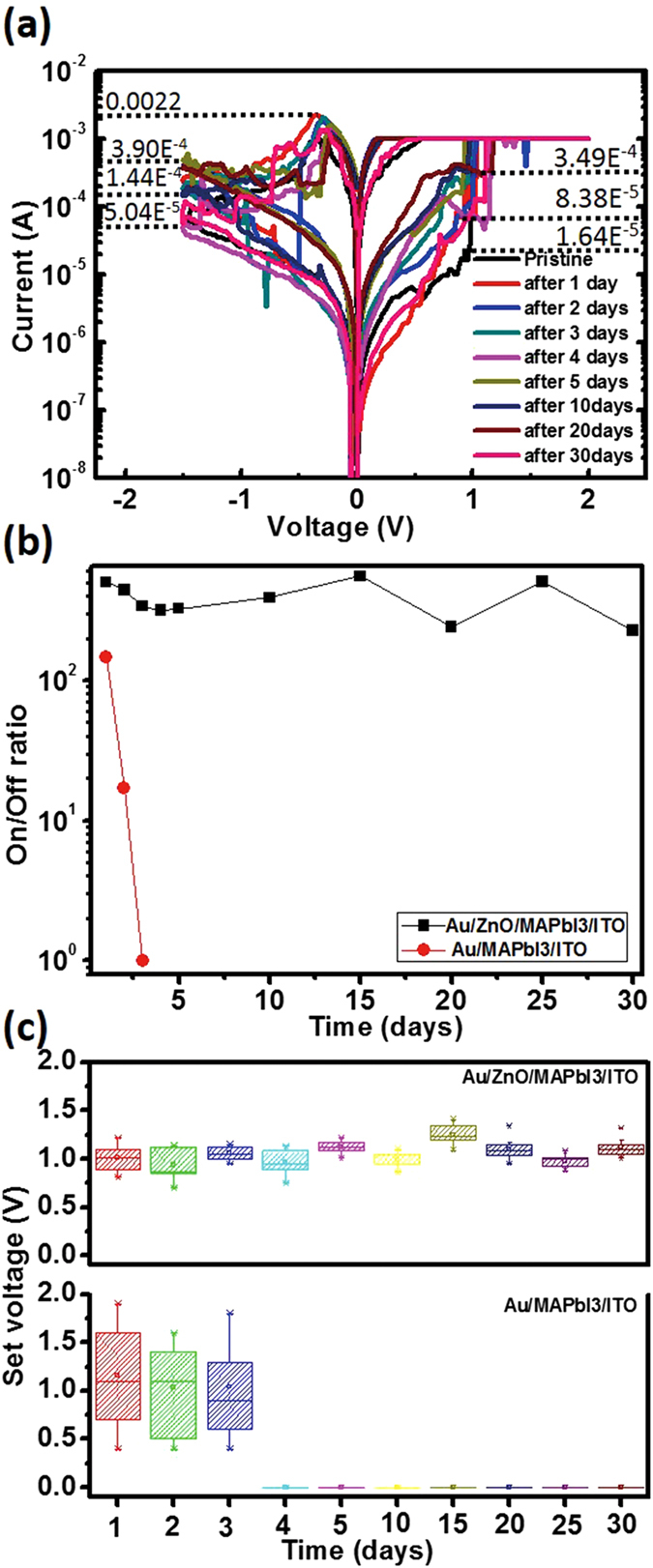
(**a**) Stable resistive switching behavior of Au/ZnO/CH_3_NH_3_PbI_3_/ITO/glass devices irrespective of storage time in an ambient atmosphere without encapsulation. (**b**) On/Off ratio and (**c**) statistical distribution of set voltages of Au/ZnO/CH_3_NH_3_PbI_3_/ITO glass and Au/CH_3_NH_3_PbI_3_/ITO devices vs. storage time in ambient air.

For the device with the ZnO layer, the electrical characteristics of Au/ZnO/CH_3_NH_3_PbI_3_/ITO devices as a function of storage time were measured in an ambient atmosphere at 23–25 °C and with 50–60% humidity using the same voltage sweep and measurement method (Fig. 3a). In this encapsulated device, the *I* − *V* curves showed bipolar resistive switching under *CC* = 1 mA after exposure to air for 30 d. During storage in air, *V*_set_ remained between 0.93 V and 1.15 V. The Au/ZnO/CH_3_NH_3_PbI_3_/ITO devices retained constant *I*_ON_/*I*_OFF_ for up to 30 d (Fig. 3b), but Au/CH_3_NH_3_PbI_3_/ITO (i.e., no ZnO layer) degraded rapidly, with a drop in *I*_ON_/*I*_OFF_ after 3 d. The variation of set voltages was selected to check the degradation of the devices (Fig. 3c). *V*_set_ varied little in the devices that were passivated by a ZnO layer remained small during storage in air for 30 d, but varied widely between 0.6 V and 1.8 V in the devices without ZnO layer after only 3 d. The degradation in the devices without the ZnO layer may be due to decomposition of CH_3_NH_3_PbI_3_ to CH_3_NH_3_I and PbI_2_. Continuous exposure to ambient air causes CH_3_NH_3_I to decompose to CH_3_NH_2_ and HI^30^; this process causes the CH_3_NH_3_PbI_3_ structure to collapse and thereby prevents formation of iodide-vacancy filaments. Encapsulation of CH_3_NH_3_PbI_3_ in a ZnO layer improved its stability in air. Thus, ZnO functions as a permeation barrier against water and oxygen due to scavenging effects such as by TiO_2_^22, 31^. Water molecules dissociate by autocatalysis on a ZnO surface^32^, and by defect-mediated dissociation on crystalline TiO_2_^33^. We assume that the scavenging effect of ZnO is a result from water dissociation, which leads to protection from moisture.

We also examined the stability of the memory device encapsulated in an AlO_x_ layer that was deposited by ALD (Al/AlO_x_/CH_3_NH_3_PbI_3_/ITO-coated glass; Figure S2a). The AlO_x_ layer was used to prevent reaction between perovskite film and Al electrode. The device with AlO_x_ showed *I* − *V* curves that retained bipolar resistive switching under CC = 1 mA after exposure to air for 30 d. During storage in air, *V*_set_ remained ~1.05 V; the stability occurs because AlO_x_ layer acts as a permeation barrier against oxygen and moisture. The electrical characteristics of Al/AlO_x_/CH_3_NH_3_PbI_3_/ITO as a function of storage time were also measured in an ambient atmosphere (Figure S3c) The Al/AlOx/CH_3_NH_3_PbI_3_/ITO devices remained constant *I*_*ON*_/*I*_*OFF*_ for up to 30 d, but without the AlO_x_ layer, *I*_*ON*_/*I*_*OFF*_ decreased rapidly after 2 d. The decrease in *I*_*ON*_/*I*_*OFF*_ after 5 d with the AlOx layer might be due to its thinness.

The variation of set voltages was measured to confirm the degradation of the devices (Figure S3d). In devices that were passivated by an AlO_x_ layer, *V*_set_ varied little during storage in air for 30 d, but in devices without the AlO_x_ layer *V*_set_ varied between 0.5 V and 1.2 V after only 2 d.

**Figure 4:**
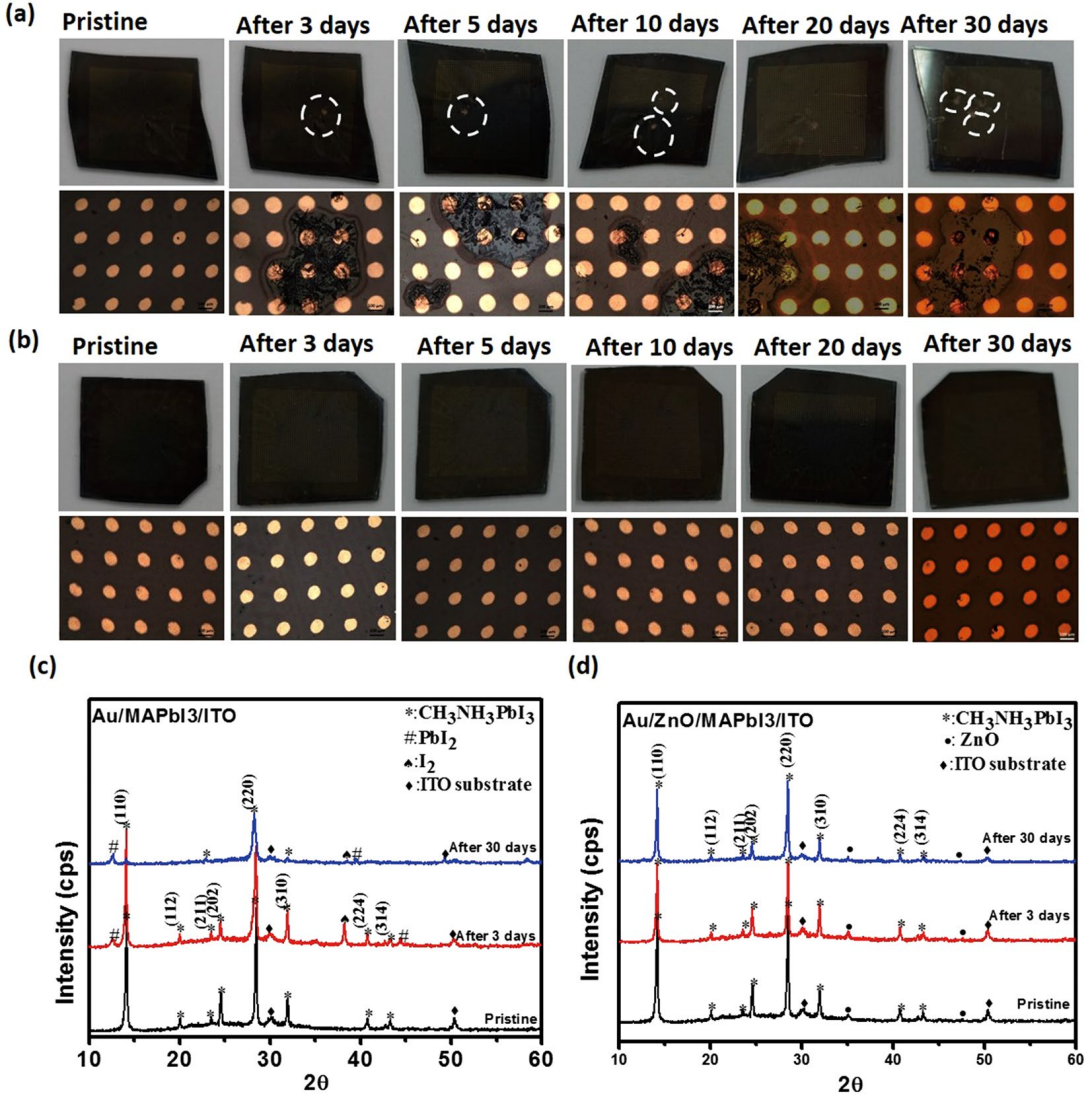
Schematic of (**a**) Au/CH_3_NH_3_PbI_3_/ITO devices and (**b**) Au/ZnO/CH_3_NH_3_PbI_3_3/ITO devices after storage in ambient air. XRD patterns of (**c**) Au/CH_3_NH_3_PbI_3_/ITO devices and (**d**) Au/ZnO/CH_3_NH_3_PbI_3_/ITO devices. Lines offset for clarity.

Images of perovskite film without (Fig. 4a) and with (Fig. 4b) encapsulated ZnO layer after storage in air (Fig. 4a,b) demonstrate that the device with ZnO layer did not degrade, whereas the device without the ZnO layer started to degrade after 1 d; they developed areas (Fig. 4b, white dotted circles) that did not exhibit resistive switching memory, and eight of the 10 devices tested showed conducting behavior due to degradation of CH_3_NH_3_PbI_3_. The XRD spectra of Au/CH_3_NH_3_PbI_3_/ITO (Fig. 4c) changed over time, but those of Au/ZnO/CH_3_NH_3_PbI_3_/ITO (Fig. 4d) did not. In the pristine state, both devices exhibited strong diffraction peaks at 14.18°, 28.48° and 31.96°, which correspond to the (110), (220) and (310) planes, respectively of tetragonal CH_3_NH_3_PbI_3_^22, 34^. In the device without the ZnO layer, XRD spectra started to form additional peaks over time due to the degradation of CH_3_NH_3_PbI_3_ in moisture. After 3 d in ambient atmosphere, the spectrum showed a peak at 2θ = 12.76° that is related to PbI_2_^35^, and a peak at 38.18° that is related to the (201) plane of I_2_^36^; these changes are due to the degradation of CH_3_NH_3_PbI_3_ by moisture. After 30 d, the film deteriorated and the main peak (110) of perovskite decreased, and another peak 39.63° that is related to the (110) plane of PbI_2_ appeared^37^. This degradation can explain the immediate malfunction of the device without passivation layer. In the device with the ZnO layer, the initial XRD spectrum showed additional diffraction peaks at 34.98° and 47.6° that can be assigned to the (002) and (102) planes of ZnO^35^. The spectrum of this device did not change noticeably after storage for 30 d at 50–60% relative humidity.

## Conclusion

We used solution-processed ZnO and ALD AlO_x_ as passivation layers under perovskite, and achieved Au/ZnO/CH_3_NH_3_PbI_3_/ITO and Al/ALD_AlO_x_/CH_3_NH_3_PbI_3_/ITO resistive switching devices that retain stable bipolar resistive switching properties in ambient air for 30 d. Devices without the passivation layer malfunctioned immediately upon exposure to air. The ZnO and AlO_x_ layer provide long-term stability of devices by protecting CH_3_NH_3_PbI_3_ from damage by humidity. Devices encapsulated with ZnO maintained their initial low operation voltage (~0.9 V) and stable *I*_ON_/*I*_OFF_ ratio after storage in air for 30 d. The devices passivated with AlO_x_ retained their pristine state after 30 d in air. These results suggest a useful and simple fabrication method that can be applied to hybrid OIP based devices to achieve practical memory devices.

## Experimental section

### Synthesis of ZnO nanoparticles

ZnO NPs were synthesized as described previously^29^. Briefly, a solution of KOH in methanol was added drop wise to a solution of zinc acetate dihydrate containing methanol with continuous stirring at 60 °C. After the reaction, the solution was washed with methanol. The upper part of the mixture was discarded after 30 min, then replaced methanol and the solution was stirred for 5 min; this process was repeated twice. Methanol was removed from the precipitated product to prevent decomposition of the perovskite layer. The NPs had diameter ~10–20 nm.

### Precursor solution preparation and device fabrication

The CH_3_NH_3_PbI_3_ layer was using a two-step spin-coating method. Under stirring at 70 °C, PbI_2_ (460 mg/ml) and MAI (50 mg/ml) were dissolved in N,N-dimethylformamide (DMF) and 2-propanol. ITO/glass substrate was cleaned with isopropyl alcohol, and deionized water, then treated using UV/O_3_ (wavelength = 253.7 nm and 184.9 nm). The PbI_2_ layer was spin-coated first at 6000 rpm for 35 s, then dried on a hotplate at 70 °C. Then CH_3_NH_3_I was spin-coated at 6000 rpm for 35 s, then the film was annealed at 100 °C for 2 h in ambient atmosphere^28^. ZnO NPs dissolved in chlorobenzene were deposited on the CH_3_NH_3_PbI_3_ layer by controlling the spin speed. Finally. dot-shaped Au electrodes were deposited on the perovskite layer by evaporation through a shadow mask.

In the Al/ALD_AlO_x_/CH_3_NH_3_PbI_3_/ITO structure, the AlO_x_ was deposited using ALD at 100 °C, which is lower than generally-used temperature (300 °C) because of the low thermal stability of CH_3_NH_3_PbI_3_. The metal precursor was trimethylaluminum (TMA) and the oxidation source was ozone^20, 38^. The deposition cycle is composed of a metal precursor pulse, N_2_ purge, ozone pulse, and N_2_ purge. The pulse and purge times during one cycle were 1.5 s TMA pulse, 1.5 s purge, 10 s ozone pulse and 3 s purge^39^. Then, dot-shaped Al electrodes were deposited on the perovskite layer by e-beam evaporation.

### Perovskite characterization

Morphological images of surface and cross section were captured using high-resolution FE-SEM (JEOL) with 10-kV acceleration voltage. Crystal structure was measured using XRD (Rigaku D/MAX-2500) with Cu Kα radiation at a step size of 0.02°. Current-voltage characteristics were measured using a Keithley 4200 in the probe station at ambient atmosphere; the voltage was controlled by one of the Au electrodes under dc sweeping voltage applied as 0 V → 2 V → 0 V → −1.5 V → 0 V, and the bottom electrode (ITO) was grounded.

## Electronic supplementary material

Supplementary Info
