# Supplementary material for: Hybrid Organic-Inorganic Perovskite Memory with Long-Term Stability in Air
Source: Sci Rep. 2017 Apr 6;7:673. doi: 10.1038/s41598-017-00778-5 (PMC5429663; doi:10.1038/s41598-017-00778-5)
Supplement: Supplementary file 1 — Supplementary Info [file 41598_2017_778_MOESM1_ESM.pdf]

Supplementary Information

Hybrid Organic-Inorganic Perovskite Memory with Long-Term Stability in Air

Bohee Hwang and Jang-Sik Lee*

Department of Materials Science and Engineering, Pohang University of Science and Technology (POSTECH), Pohang 790-784, Republic of Korea

*e-mail: jangsik@postech.ac.kr

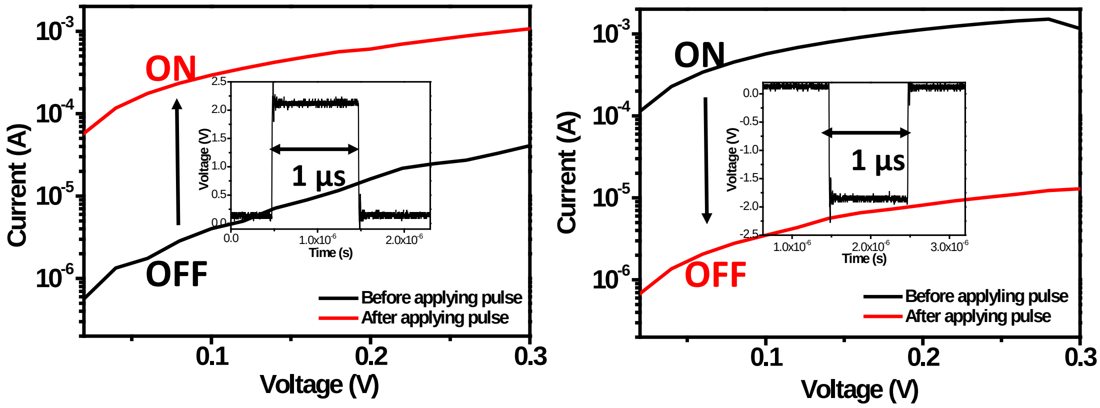

Figure S1. (a) I-V curves of the OFF and the ON states of the Au/$CH_3NH_3PbI_3$/ITO device before and after applying the voltage pulse with 2 V and 1μs at set process, (b) Reset process by a voltage pulse with -2 V and 1μs. (Inset:applied pulse)

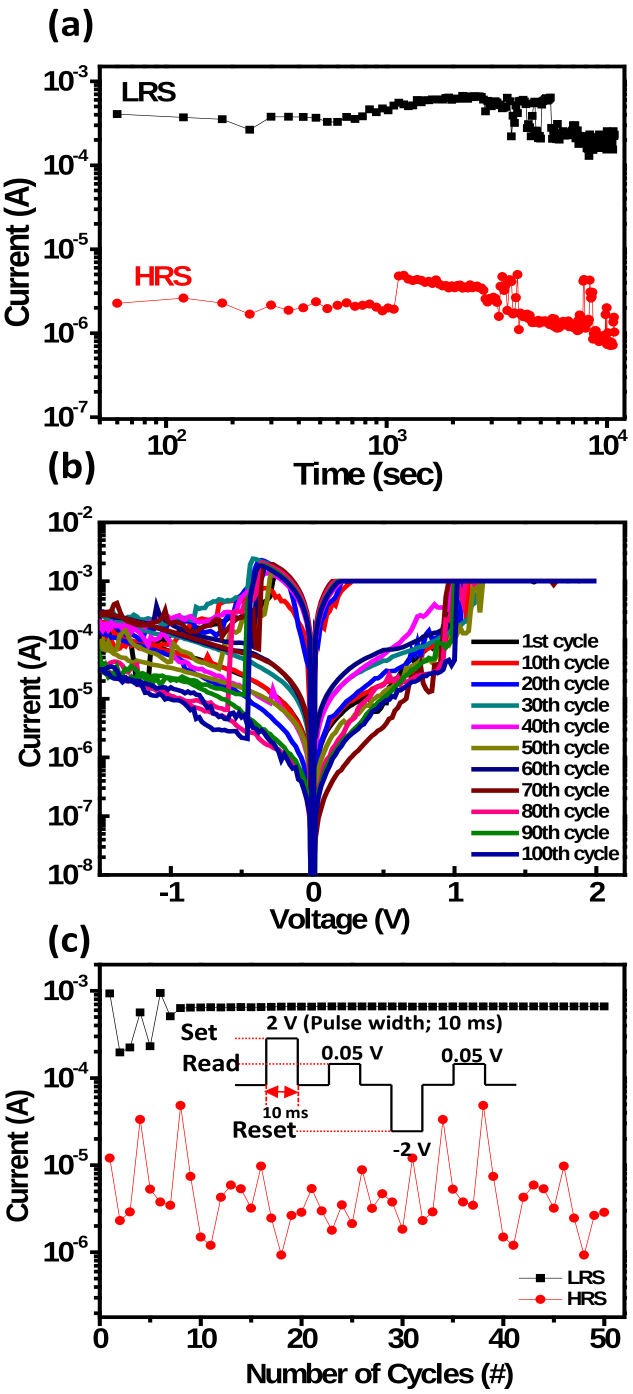

Figure S2. Resistive switching of Au/ZnO/$CH_3NH_3PbI_3$/ITO structures. (a) Data retention characteristics of LRS and HRS states at room temperature. (b) Endurance of Au/ZnO/$CH_3NH_3PbI_3$/ITO device with stable switching for 100 cycles.

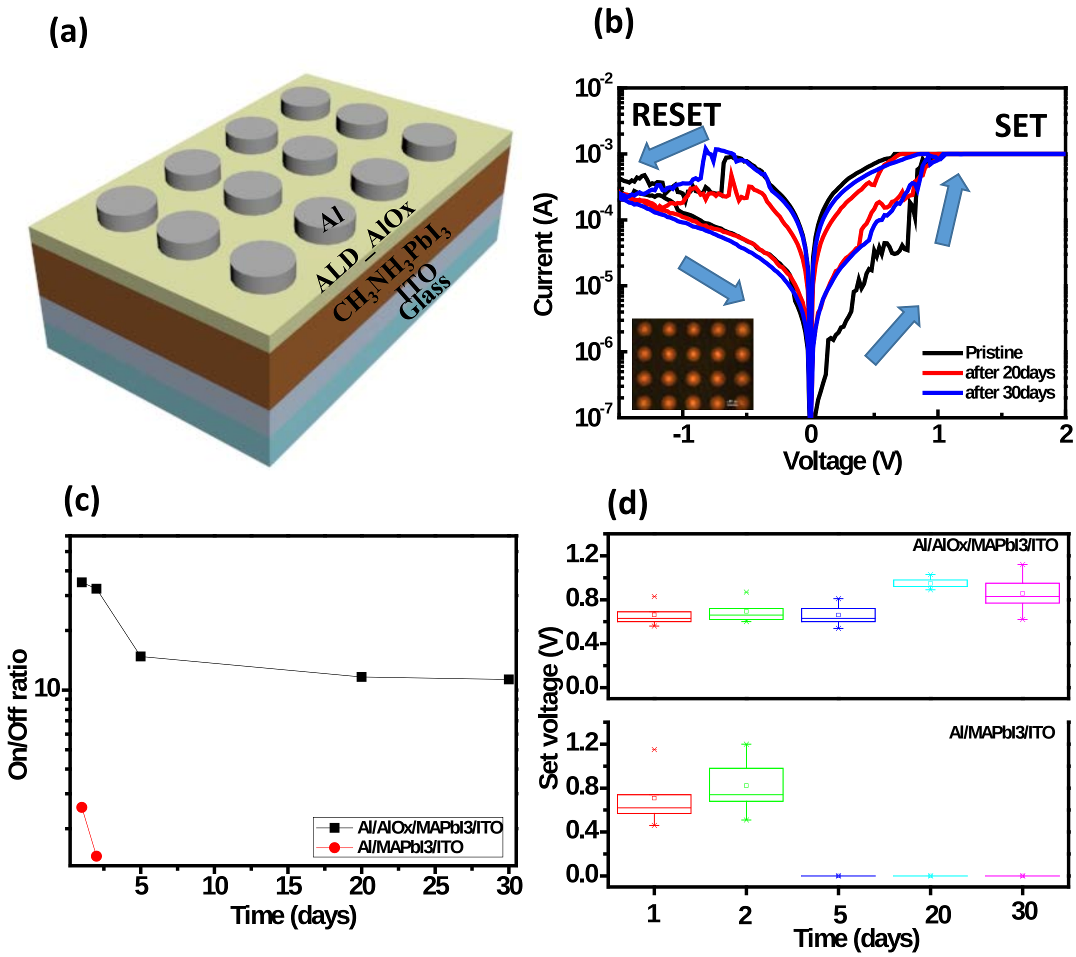

Figure S3. Schematics of (a) Al/AlO_x/$CH_3NH_3PbI_3$/ITO devices. (b) Resistive switching characteristics of the AlO_x-encapsulated perovskite device over storage time in ambient air; inset: top view of memory device.
